# RESEARCH ON THE IMPACT OF INTERGENERATIONAL DEMOGRAPHIC EVOLUTION ON COMMUNITY SERVICES IN CHINA (2024–2050)

**DOI:** 10.1093/geroni/igae098.3534

**Published:** 2024-12-31

**Authors:** Yuxia Wu, Wanwan Si, Hong Mi

**Affiliations:** Ningbo University of Technology, Ningbo, Zhejiang, China (People’s Republic); Zhejiang University, Hangzhou, Zhejiang, China (People’s Republic); Zhejiang University, Hangzhou, Zhejiang, China (People’s Republic)

## Abstract

Facing the dual challenges of population aging and a declining birthrate, China is actively promoting mutual assistance and support among multiple generations through a combination of policies and coordinated services. Utilizing datasets from multiple Chinese censuses and the China Family Panel Studies (CFPS), this study employs microsimulation models to establish predictive frameworks for both population intergenerational shifts and service demands. It delves into the trajectories of generational transitions and their subsequent impact on community-based services over the forthcoming 25 years in China. Building upon the long-term care practices and experiences in generational integration within OECD nations, the study proposes phased service policy alignments and synergistic strategies.The research underscores that defining parameters such as demographic structures, health statuses, care paradigms, and multi-generational dynamics, along with segmenting service recipients and fine-tuning policies and service collaborations, enables precision targeting and flexible adjustments in community services. This, in turn, fosters “upward intergenerational” connections — where individuals in their early senior years provide informal caregiving to those who are significantly older and may have physical limitations — and “downward intergenerational” bonds — in which grandparents contribute to the upbringing of their grandchildren. Such arrangements not only reinforce societal bonds but also alleviate the caregiving responsibilities of families, particularly those with members in their mid-30s to mid-40s, who often juggle between child-rearing and older adults care responsibilities.

